# Efficacy of an Anthocyanin and Prebiotic Blend on Intestinal Environment in Obese Male and Female Subjects

**DOI:** 10.1155/2018/7497260

**Published:** 2018-09-13

**Authors:** Shelly N. Hester, Angela Mastaloudis, Russell Gray, Joseph M. Antony, Mal Evans, Steven M. Wood

**Affiliations:** ^1^Pharmanex Research, NSE Products Inc., 75 West Center Street, Provo, UT 84601, USA; ^2^KGK Science Inc., 255 Queens Avenue, Suite 1440, London, Ontario, Canada N6A 5R8

## Abstract

**Background:**

Anthocyanins and prebiotics impact overall health and wellness, likely through modulation of the microbiota and the intestinal ecosystem.

**Objectives:**

An 8-week open-label study in male and female volunteers with uncomplicated obesity was designed to study the efficacy of an anthocyanin and prebiotic blend in modulating intestinal microbiota and intestinal inflammation.

**Results:**

After 8 weeks of daily supplementation, participants had a significant decrease in Firmicutes (*p* < 0.001) and Actinobacteria (*p* < 0.001) and a significant increase in Bacteroidetes (*p* < 0.001). Bowel habits were improved as evidenced by reductions in the severity of bloating (*p* < 0.05), gas (*p*=0.035), and abdominal pain (*p*=0.015) as well as significant improvements in stool consistency (*p* < 0.05). Finally, a nonsignificant decrease in the inflammatory marker fecal calprotectin was seen (*p*=0.107). The supplement was safe and well tolerated.

**Conclusions:**

The results suggest that regular consumption of the anthocyanin-prebiotic blend positively modulated the intestinal ecosystem and provided insights into the mechanisms of action and its impact on health benefits.

## 1. Introduction

Anthocyanins and prebiotics have been shown to impact overall health and wellness [1–3]. One mechanism for the health benefits from these ingredients may be through modulation of the microbiome and the intestinal ecosystem and merits further investigation. The intestinal ecosystem is a complex network of bacterial cells, host cells, cellular mediators, and tissues. The relative abundance of two groups of bacteria that are dominant in the gut—Bacteroidetes and Firmicutes—is associated with overnutrition, high-fat diet, and obesity, with the Bacteroidetes population decreasing and Firmicutes population increasing with these conditions [4, 5]. It has been demonstrated that the relative proportion of Bacteroidetes is decreased in obese people by comparison with lean people and the Bacteroidetes proportion increases with weight loss [6]. This change in the function and composition of the gut microbiota (increased Firmicutes : Bacteroidetes ratio) represents a dysbiotic environment characterized by increased systemic and gastrointestinal tract inflammation that has adverse consequences. It could be that bacterial species promoting obesity-associated inflammation belong to the Firmicutes and that microbiota associated with nonobese population may have a protective effect [7]. Studies described by Gordon and colleagues raise the possibility that the gut microbiota plays a significant role in contributing to differences in body weight among individuals [6, 8]. The results obtained from earlier studies show that obesity alters the nature of the intestinal microbiota and also that differences in the efficiency of caloric extraction from food may be dependent on the microbiota, which in turn may contribute to obesity [9]. An obese population was used in the current study because the microbial composition has been shown to be different between lean and obese individuals, as well as individuals consuming different compounds, including anthocyanins and prebiotics [6, 10].

Anthocyanins are a unique subgroup of flavonoids that have been demonstrated to beneficially impact the microbiota [11–13], along with exerting anti-inflammatory effects [14, 15]. Both clinical and *in vitro* experiments have demonstrated the beneficial properties of anthocyanins on microbiota [11, 12, 16]. Current evidence indicates that when consumed, anthocyanins are present throughout the length of the gastrointestinal tract including the colon, where gut microbiota may play a crucial role in their bioactivity, and conversely, anthocyanins may positively influence the balance of the microbiota [17–19]. Additionally, research has demonstrated that anthocyanins seem to have an antiobesity effect that may be connected to their modulation of the microbiota [10]. These antiobesity effects include preventing body fat accumulation, insulin insensitivity, inflammation, and dyslipidemia [20].

Prebiotics, including short-chain fructooligosaccharides (scFOSs) and inulin included in the anthocyanin-prebiotic blend evaluated in the current study, have been demonstrated to be beneficial to intestinal health. The scFOS (degrees of polymerization (DP) 2–4) is a fiber that is fermented rapidly in the proximal part of the large intestine, while inulin (average DP ≥ 10) is fermented more slowly and is therefore able to reach the more distal parts of the large intestine. Supplementation of prebiotics with a range of DP allows maximal effects throughout the entire intestinal tract. Many studies have demonstrated scFOSs and inulin's positive effects on bacterial populations and markers of intestinal health in humans [21–27].

To our knowledge, no previous study has measured the combined effects of scFOSs, inulin, and anthocyanins on human gut microbiota and intestinal inflammation. The hypothesis for this study was that the anthocyanin and prebiotic blend would improve intestinal health. The objective of this open-label study was to assess the intestinal benefits of an anthocyanin-prebiotic blend in adults with uncomplicated obesity, including microbiome, bowel habits, and intestinal inflammation.

## 2. Materials and Methods

### 2.1. Study Design

This study was reviewed by the Therapeutic Products Directorate (TPD) and the Natural and Non-prescription Health Products Directorate (NNHPD) of Health Canada, and approvals were obtained on March 1, 2016, from the TPD, Ottawa, Ontario. Research ethics board approval was obtained on March 14, 2016, from Institutional Review Board Services, Aurora, Ontario. This study was conducted in accordance with the ethical principles that have their origins in the Declaration of Helsinki and its subsequent amendments (clinicaltrials.gov identifier NCT02743195).

**Figure 1:**
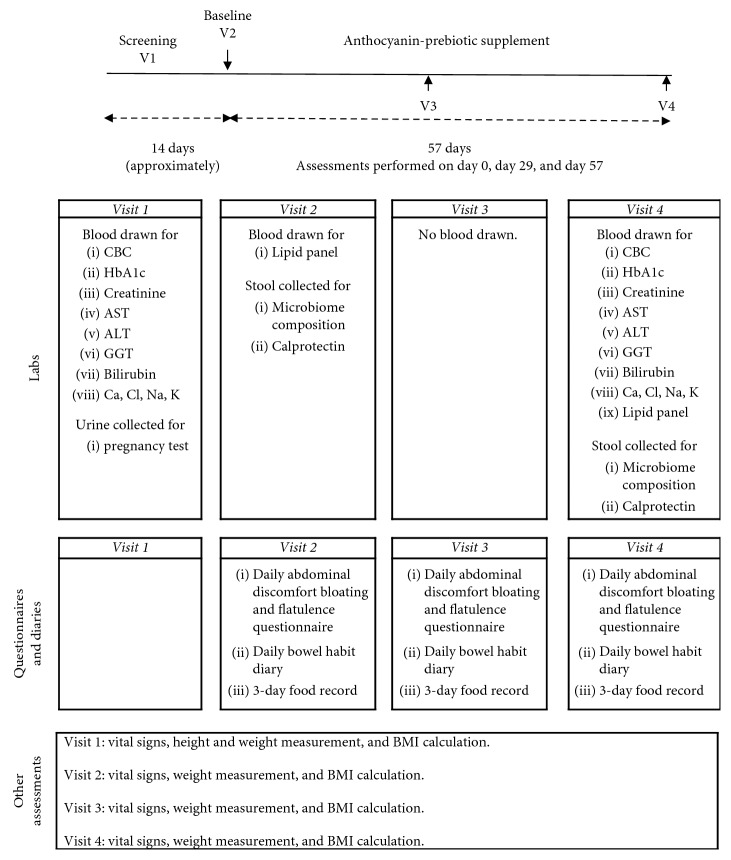
Study flow diagram. Abbreviations used: complete blood count (CBC), hemoglobin A1c (HbA1c), aspartate aminotransferase (AST), alanine aminotransferase (ALT), gamma-glutamyl transferase (GGT), calcium (Ca), chloride (Cl), sodium (Na), potassium (K), and body mass index (BMI).

This was an open-label study, conducted at a single-center, KGK Science Inc., in London, ON, Canada, between March 23, 2016, and June 14, 2016. The study was sponsored by Pharmanex Research, NSE Products, Inc., Provo, UT. The duration of this study was eight weeks with three in-clinic visits: baseline, week 4, and week 8, as outlined in Figure 1. A run-in period of 14 days was initiated, and participants were instructed to begin completing daily diaries of abdominal discomfort, bloating, and flatulence questionnaire and daily bowel habits along with weekly three-day food records. Bowel habits were collected to check on any changes experienced during intervention. After eligibility was confirmed during the baseline assessments, all subjects received the anthocyanin-prebiotic blend supplementation for eight weeks (day 1 to day 57). A 3-day food record (two weekdays and one weekend day) at baseline, four weeks, and eight weeks was used to ensure diet as assessed at baseline was maintained throughout the study. Diet was analyzed using NutriBase Clinical Nutrition Manager version 7.14 (Phoenix, AZ). The 3-day food records were entered into NutriBase by nutritionists. During the subsequent 8-week period, participants were required to continue completing daily abdominal discomfort, bloating, and flatulence questionnaire and daily bowel habits diary as well as their weekly three-day food records.

### 2.2. Subjects

Recruitment for this study was performed within the region of Southwestern Ontario, Canada, using KGK Science Inc.'s internal participant database along with local electronic and physical advertisements, with no sex or racial bias. The inclusion criteria were as follows: males and females between the ages of 18 and 50 years (inclusive); if female, either not of child-bearing potential or using a medically approved method of birth control; healthy individuals as determined by laboratory results, medical history, and physical examination; a body mass index (BMI) of 29.9–39.9 ± 1 kg/m^2^; those who agreed to maintain regular diet (with the exception of avoiding pro- and prebiotics and anthocyanin-rich foods) and exercise; and voluntary written and informed consent to participate in the study.

Exclusion criteria were as follows: women who were pregnant, breastfeeding, or planning to become pregnant during the course of the study; record of gastrointestinal surgery (except appendectomy, hernia repair, or hemorrhoidectomy); previous history of gastrointestinal diseases (except hemorrhoids and uncomplicated diverticula); type 1 or uncontrolled type 2 diabetes; previous history of smoking within one year of baseline; active eating disorder; presence of rectal bleeding; recent weight loss (greater than 5 kg in the past month); use of over-the-counter or prescription laxative medication within four weeks prior to baseline; use of oral antibiotics within five weeks of baseline; use of anti-inflammatory medications four weeks prior to randomization and for the duration of the study; consumption of anthocyanin-rich foods, such as blueberries, blackberries, strawberries, and wine, during the study; not willing to discontinue the use of pre- and probiotic and/or polyphenol supplements for four weeks prior to baseline and for the duration of the study; and any other condition which, in the investigator's opinion, may have affected the subject's ability to complete the study or its measures, or may have posed a significant risk to the subject.

### 2.3. Intervention

Participants were instructed to take one sachet of powder every morning with breakfast by mixing into beverage or food of choice. The supplement delivered 215 mg anthocyanins and 2.7 g prebiotic fibers daily and consisted of anthocyanins (144 mg European blueberry extract (35 mg anthocyanins), 202 mg black currant extract (60 mg anthocyanins), and 618 mg black rice extract (120 mg anthocyanins)) and a prebiotic blend (1.9 g inulin (Orafti GR, Beneo, Tienen, Belgium) and 1.1 g fructooligosaccharides (Nutraflora, scFOS^®^, Ingredion, Westchester, IL)) (90% purity of 3.0 g delivers a total of 2.7 g prebiotic fibers daily). The supplement sachets were given to participants at baseline and 4-week visits (30 sachets given at each visit). Stability testing results showed supplement ingredients were stable throughout the study. Anthocyanins were tested with pH differential UV/Vis, a method set up by referencing the AOAC Official Method 2005.02, “Total monomeric anthocyanin pigment content of fruit juice, beverages, natural colorants, and wines.” Fiber was analyzed by Total Dietary Fiber (Codex Definition) by Medallion Labs (Minneapolis, MN).

### 2.4. Outcome Measures

The primary outcome of this study was the change in fecal microbial composition from day 0 to day 57 as a result of supplementation with an anthocyanin-prebiotic blend. This was quantified from fecal samples obtained from participants who were provided with an EasySampler® Stool Collection Kit (GP Medical Devices, Holstebro, Denmark) containing materials required to collect their stool sample within 48 h prior to their baseline and the end of study visits. The stool samples were frozen and transported to the clinic with ice packs to ensure the samples remained frozen. For sample preparation, 150 mg fecal samples were placed into a 2 mL microfuge tube. DNA was extracted with the Qiagen QIAamp DNA Stool Mini Kit prior to analysis. Fecal microbial composition was measured by 16S rRNA sequencing on the Illumina platform (San Diego, CA) at Reed Research Group Laboratory at University of Wisconsin. Quantitative Insights Into Microbial Ecology (QIIME) analysis was done [28]. The reads from the sequencer were filtered, and operational taxonomic units (OTUs) were generated. Specifically, alpha-diversity, beta-diversity, and relative abundance of the most represented phyla (Actinobacteria, Bacteroidetes, and Firmicutes) were measured from these sequences. Sequences were aligned with PyNAST to generate alpha-diversity plots and beta-diversity plots with UniFrac.

Levels of fecal calprotectin were measured by Human S100A8/S100A9 Heterodimer DuoSet ELISA (R&D Systems, Minneapolis, MN; Catalog no. DY8226-05) as per the manufacturer's protocol at Reed Research Group Laboratory at University of Wisconsin. The frozen fecal samples were diluted 1 : 2 in phosphate-buffered saline (PBS) (pH 7.2) containing a protease and phosphatase inhibitor (Thermo Fisher Scientific; Catalog no. 78440). The samples were centrifuged at 10,000 ×g at 4°C for 15 minutes. Supernatants were then filtered through a 0.45 *µ*m polyethersulfone (PES) membrane syringe filter and stored at −80°C until analyzed by ELISA [29, 30].

Anthropometric measurements were taken, including height (screening visit), weight (all visits), and BMI (all visits).

Participants were required to complete a daily bowel habits diary, an abdominal discomfort, bloating, and flatulence questionnaire, and the Bristol Stool Scale (BSS) [31] commencing seven days prior to baseline and through the end of the study.

A secondary outcome of the study was the change in hemoglobin A1c (HbA1c) which was measured at baseline and end of the study. Whole blood was collected into 4 mL EDTA tubes and then analyzed on the cobas c 513 analyzer (Roche Diagnostics) at LifeLabs in Toronto, ON, Canada.

Safety was assessed by the complete blood count (CBC), electrolytes, creatinine, aspartate aminotransferase (AST), alanine aminotransferase (ALT), gamma-glutamyl transferase (GGT), bilirubin, and lipid panel including total-cholesterol, LDL-C, HDL-C, and triglycerides and adverse event reporting.

### 2.5. Sample Size

The planned sample size for this study was 51 participants, with at least 25% gender representation. Power calculations were performed to determine the required sample size to provide 80% power at the 0.05 alpha level and an attrition rate of 25% when comparing changes in microbial composition from baseline to day 57.

### 2.6. Compliance

Compliance was assessed by counting the returned study product at each visit. Percent compliance was calculated by determining the number of dosage units consumed divided by the number expected to have been taken and multiplied by 100%. In the event of a discrepancy between the information in the subject diary and the amount of the study product returned, calculations were based on the product returned unless an explanation for a lost product was provided. Participants found to have a compliance of <80% or >120% at any visit were counseled. Compliance of <70% or >130% was considered as noncompliant, and any subject demonstrating noncompliance for two consecutive visits was withdrawn from the study.

### 2.7. Statistical Analysis

Numerical efficacy endpoints were formally tested for significance by the paired Student's *t*-test. Significant efficacy of the supplement was inferred if the changes from baseline were significantly different from zero (*p*≤0.05). Numerical endpoints that were intractably nonnormal were assessed by the nonparametric signed-rank test. Probabilities ≤0.05 were considered statistically significant, and probabilities ≤0.10 were considered trends. All statistical analyses were completed using the R Statistical Software Package version 3.2.2 (R Core Team, 2015) for Microsoft Windows.

## 3. Results

### 3.1. Participant Baseline Characteristics

Fifty-one volunteers were enrolled into the open-label study. Thirty-four female and 12 male volunteers, aged 42.9 ± 10.0 years and with BMI 34.2 ± 3.1 kg/m^2^, for a total of 46 participants completed the study (Table 1). The 46 participants included in the analysis demonstrated an overall product compliance of 97.7%.

### 3.2. Anthropometrics

Weight did not significantly change during the study with a weight of 94.8 ± 13.1 kg, 95.1 ± 13.0 kg, and 94.9 ± 12.8 kg at baseline, 4 weeks, and 8 weeks, respectively. Additionally, the BMI did not significantly change during the study with a BMI of 34.0 ± 3.1 kg/m^2^, 34.1 ± 3.1 kg/m^2^, and 34.0 ± 3.2 kg/m^2^ at baseline, 4 weeks, and 8 weeks, respectively.

### 3.3. Diet

There were no statistically different changes in the average daily total caloric intake, fiber intake, and macronutrient profile of participants who were supplemented with the anthocyanin-prebiotic blend at baseline compared to week 4 and week 8 (Table 2). However, riboflavin (vitamin B_2_), a micronutrient, and sodium, a mineral, showed statistically significant differences. Intake of daily riboflavin decreased by 0.19 ± 0.52 mg from baseline to week 4 (*p*=0.018), and intake of sodium decreased by 334 ± 1082 mg from baseline to week 8 (*p*=0.043).

### 3.4. Effects on Gut Microbiome Composition

**Figure 2:**
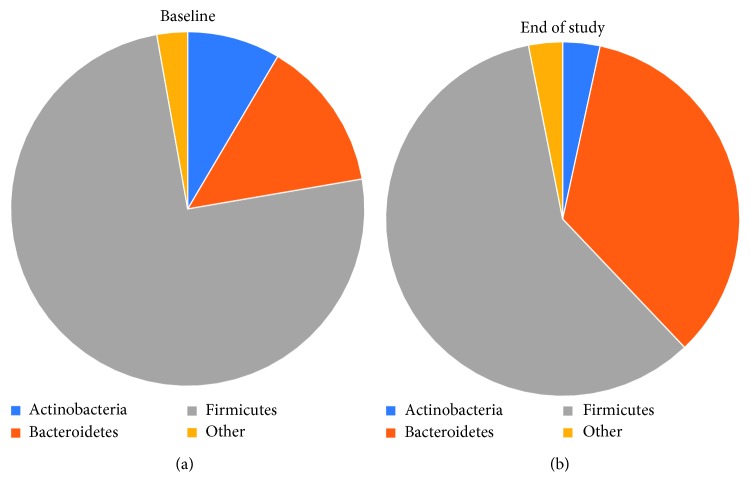
Composition at the phyla level shown for (a) baseline and (b) end of the study.

**Figure 3:**
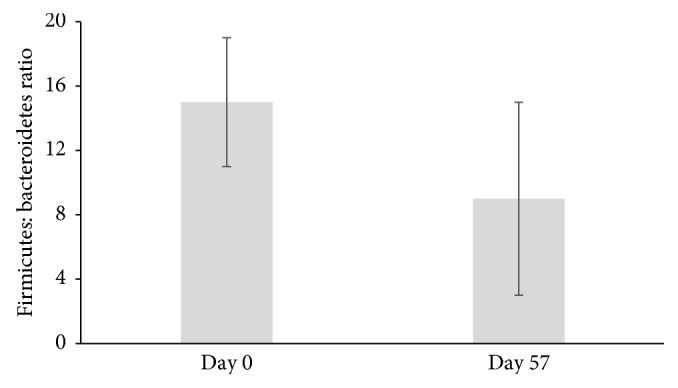
Ratio of Firmicutes to Bacteroidetes in participants supplemented with the anthocyanin-prebiotic blend at baseline (day 0) and end of the study (day 57) (*n*=45); *p* < 0.001 (mean ± SD).

**Figure 4:**
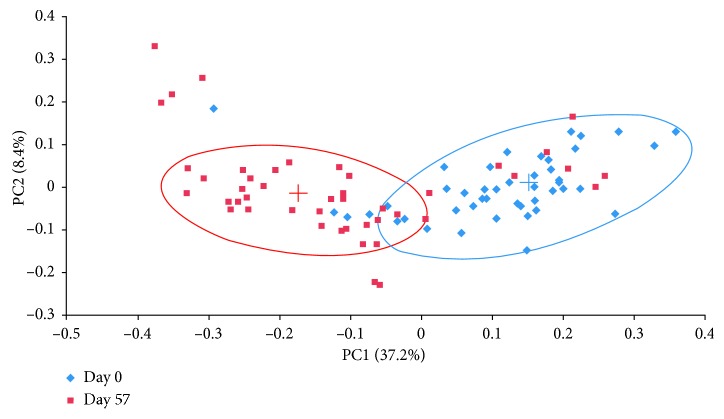
Scatterplots of the first two principal components of the weighted UniFrac beta-diversity measure at day 0 (blue) and day 57 (red). Cluster centroids were found using K-means (blue cross is day 0 and red cross is day 57) clustering for two clusters. Approximate clusters are encircled (*n*=45).

After 8 weeks of daily supplementation, participants had a significant decrease in Firmicutes (*p* < 0.001), Actinobacteria (*p* < 0.001), and Firmicutes-to-Bacteroidetes ratio (*p* < 0.001) and a significant increase in Bacteroidetes (*p* < 0.001). The composition at the phyla level is demonstrated in Figure 2. Firmicutes decreased from 74.9% to 59% (*p* < 0.001), and Bacteroidetes increased from 13.8% to 34.5% (*p* < 0.001) (Table 3) over the course of the study. The ratio of Firmicutes : Bacteroidetes decreased from 14.2 to 9.3 (*p* < 0.001) (Figure 3) (Firmicutes : Bacteroidetes ratio for individual participants was determined, and then, the average ratio was calculated). Actinobacteria decreased from 8.5% to 3.4% (*p* < 0.001) (Table 3). These three phyla made up 97.2% of the bacterial composition at baseline and 96.9% following 8-week supplementation. The diversity and richness, as examined by alpha-diversity, of the samples were unaffected by the supplement. Weighted UniFrac beta-diversity that measured species abundance and weight branch length with an abundance difference changed from 0.384 ± 0.067 to 0.361 ± 0.055 following supplementation but was not significantly different. Principal coordinate analysis (PCoA) of UniFrac distances was performed to compare the data set before and after intervention. The PCoA plot using weighted UniFrac distances showed a completely different profile (Figure 4). All samples at baseline tended to show similar overall phylogenetic trees that were different at the end of the study. The gut microbial community of each group member was clustered, and the principal coordinate accounted for 37.21% of the variance.

### 3.5. Effects on Fecal Calprotectin

**Figure 5:**
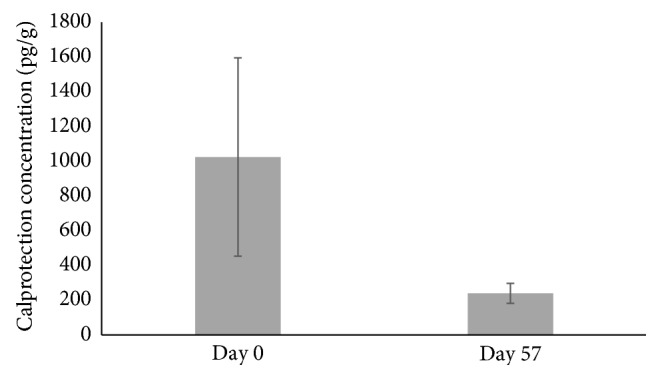
Fecal calprotectin concentration in participants supplemented with the anthocyanin-prebiotic blend at baseline (day 0) and end of the study (day 57) (*n*=43); *p*=0.107 (mean ± SEM).

A nonsignificant reduction in the concentration of fecal calprotectin was found after participants consumed the supplement for 8 weeks (*p*=0.107), in intestinal inflammation (Figure 5) (*n*=43; one subject was unable to provide a fecal sample at the end of the study and two fecal samples were not available for calprotectin analysis).

### 3.6. Effects on HbA1c

HbA1c significantly decreased from 5.51 ± 0.37 mmol/L at baseline to 5.35 ± 0.39 mmol/L at the end of the study (*p* < 0.001). Four participants with elevated HbA1c were prediabetic and one participant was diabetic when enrolled into the study; after supplementation, these participants showed a normalization or reduction in HbA1c levels.

### 3.7. Effects on Abdominal Discomfort and Stool Consistency

There was a significant increase of 5% in the Bristol Stool Scale (BSS) at week 7 (*p*=0.028) compared to baseline, suggesting participants had a more normal bowel consistency with a score of four on the BSS (scale 1–7; 1 = very hard and 7 = entirely liquid) after supplementation, but there was a trend towards statistical significance at week 6 (4%; *p*=0.051) and week 8 (5%; *p*=0.063) (Table 4). No significant changes were found in the number of bowel movements at any time point when compared to baseline. However, participants reported significant improvements in their bowel habits at weeks 4 through 8, with significant reductions in the proportion of bowel movements that were straining to start when compared to baseline (Table 4). The proportion of incomplete bowel movements was significantly reduced from week 2 through the end of the study (*p* < 0.001) when compared to baseline. These improvements in bowel habits support the significant improvements in the BSS reported by participants. Furthermore, there was a significant reduction in the severity of bloating and abdominal pain (*p* ≤ 0.050) for weeks 3 through 8 when compared to baseline (Table 5). Participants also reported significantly less gas severity at week 5 (*p*=0.007), week 7 (*p*=0.043), and week 8 (*p*=0.035) when compared to baseline.

### 3.8. Safety

No changes in vital signs and blood parameters measured were deemed clinically significant by the investigator.

Thirty adverse events (AEs) were recorded by 18 participants over the duration of the study. Of the 30 reported AEs, only 2 events were considered as having a probable, and ten as a possible, relation to supplementation. The rest of the adverse events were assessed as unlikely related or not being related to supplementation. The two probable AEs, and their total occurrences, were fecal discoloration (1) and tooth discoloration (1), which would be expected from the color of the anthocyanins in the supplement. The ten possible AEs, and their total number of occurrences, were abdominal discomfort (1), diarrhea (1), fecal discoloration (4), frequent bowel movements (1), and vomiting (3). The supplement contained fibers which have been reported in previously published clinical studies to cause gastrointestinal complaints and adverse symptoms, but symptoms have been reported to dissipate with continued use. In comparison with probiotic and prebiotic studies in overweight and obese adults where 67% of the participants reported at least one potentially product-related AE, the current study showed only 16% of the participants experienced an AE that was potentially related to intervention [32]. The severity of the AEs was also reported. Nine AEs were mild, one moderate, and two severe. One mild (diarrhea) and two severe (nausea and vomiting) AEs required the discontinuation of the supplement. All other AEs were resolved by the end of the study.

## 4. Discussion

The findings of this open-label study indicate that consumption of an anthocyanin-prebiotic blend positively modulated the intestinal ecosystem. Supplementation with the anthocyanin-prebiotic blend achieved a significant reduction in the proportions of Firmicutes and a significant increase in Bacteroidetes. Associations between higher proportions of Firmicutes with obese individuals and those of Bacteroidetes with lean individuals have been reported [6, 33]. Obese individuals had higher Firmicutes and reduced Bacteroidetes than did lean controls, prior to diet therapy, wherein a low-calorie diet that comprised either fat or carbohydrate restriction led to a reduced abundance of Firmicutes and an increased abundance of Bacteroidetes [6]. Studies have consistently shown that the ratio of Firmicutes to Bacteroidetes is positively correlated with kilocalorie diet and weight loss [4, 34]. Although a lower Firmicutes-to-Bacteroidetes ratio has generally been associated with lean individuals, contradictory results have also been reported [35]. Despite contrary views, an increased presence of Bacteroidetes and a lower presence of Firmicutes are likely an advantage to the host. Body weight did not change during the course of the study. A longer-term study designed to investigate weight loss is needed to determine if a lower Firmicutes-to-Bacteroidetes ratio could decrease weight since Firmicutes are more effective at harvesting energy from foods than Bacteroidetes [8, 36]. Further research is also needed to determine if an anthocyanin-prebiotic blend could impact weight control since there is previous research demonstrating anthocyanin's antiobesity benefits in obese populations through gut microbiota interactions [10].

The decrease in Actinobacteria was not expected since previous research has shown that blueberry powder supplementation (375 mg anthocyanins and 4.2 g fiber daily, delivered in 25 g blueberry powder) increased counts of *Bifidobacterium*, which belongs to the Actinobacteria phylum [11, 12]. *Ex vivo* and *in vitro* studies have also demonstrated anthocyanin's ability to increase counts of *Bifidobacterium* and *Lactobacillus*, while decreasing bacterial populations such as *E. coli* [13, 16]. Additionally, previous research has also demonstrated that scFOSs at similar levels supplemented in the current study have significantly increased bifidobacteria [21, 23]. Additionally, a decrease in Actinobacteria phylum could also be attributed to the decrease of another genus within the phylum, such as *Actinomyces, Adlercreutzia, Collinsella, Eggerthella, and Slackia*. Although the current study cannot differentiate whether the changes in microbiota could be attributed to anthocyanins, prebiotics, or the combination, future studies are planned to answer this question and will help us better understand the reduction of Actinobacteria observed in the current study.

The current study found a nonsignificant reduction in fecal calprotectin. Neutrophils respond to inflammation in the gastrointestinal tract, where they migrate to and release calprotectin in the stool. Therefore, a decrease in fecal calprotectin concentration is a marker of reduced intestinal inflammation. Intestinal microbiota composition is associated with both local and systemic inflammation in obesity, and the short duration of the current study may have resulted in a trend in reduction of fecal calprotectin. A longer study duration may have resulted in a significant decrease in fecal calprotectin, since the positive changes in microbiota proportions may have had a larger impact on the development of low-grade inflammation [7]. The levels of fecal calprotectin at the beginning of the study were not as high as previous levels found in other obese subject populations [37–39]. Although fecal calprotectin is a test that is commonly used in research, little work has been done to determine normal levels in different populations [40].

Higher doses of inulin and scFOSs have been previously shown to induce gas, bloating, and abdominal pain severity [25, 27, 41, 42]. Abdominal health parameters were collected to check on any changes experienced during the intervention. In the current study, anthocyanins with lower levels of prebiotics mediated positive intestinal benefits, especially regarding the microbiota, but did not induce discomfort and improved severity of abdominal pain, gas, and bloating. Since participants were healthy and less likely to have experienced abdominal pain, gas, or bloating at baseline, improvement in all abdominal health parameters investigated following supplementation was noteworthy. Additionally, no dietary changes during the study were found that could explain differences in BSS parameters.

A reduction in HbA1c was experienced by participants, and this positive outcome for a marker of long-term glucose regulation is especially notable since none of these subjects experienced a substantial change in bodyweight or changes in dietary habits. Although not an expected outcome in the current study, previous research has demonstrated that berries and anthocyanins improve glucose control in individuals with both normal and impaired glucose regulation [1,43–46]. Further research is needed to better understand how the change in gut microbes may be contributing to reduction in HbA1c. However, studies have demonstrated the positive impact dietary anthocyanins have on microbiota in obese populations which then leads to obesity control, including decreased insulin resistance, decreased inflammation, and decreased fat storage [10]. Additionally, previous work from our group has demonstrated that a similar anthocyanin blend used in the current clinical study modulated inflammation and improved insulin resistance in high-fat-fed mice [47].

There are a few limitations of this study. One major limitation of this study is the absence of a control group. This study was designed as an open-label study where each subject served as their own controls. This design was implemented to identify outcomes to further investigate in the future, placebo-controlled studies. Another limitation was the accuracy of metagenomics analyses, which was not optimal since the relative abundance of specific taxa was not measured.

## 5. Conclusions

The current preliminary study confirms a role of the blend of inulin, scFOSs, and anthocyanins to change the intestinal environment in a mildly and moderately obese population. The 8-week supplementation achieved a significant reduction in the proportions of Firmicutes and a significant increase in the Bacteroidetes, with a nonsignificant reduction in levels of fecal calprotectin. The anthocyanin-prebiotic blend also improved BSS, gas, bloating, and abdominal pain severity. Overall, the results of this study suggest that regular consumption of the blend of anthocyanins and prebiotics positively modulated the intestinal ecosystem, including the microbiota, and provided insights into the mechanisms of action of the anthocyanin-prebiotic formulation and its impact on health benefits.

## Figures and Tables

**Table 1:** Participant characteristics at the beginning of the study (*n*=46).

Variable	Value
Age, mean ± SD	42.9 ± 10.0
Height (cm), mean ± SD	166.8 ± 9.4
Weight (kg), mean ± SD	94.8 ± 13.1
BMI (kg/m^2^), mean ± SD	34.0 ± 3.1
HbA1c (mmol/L), mean ± SD	5.51 ± 0.37
Systolic blood pressure (mmHg), mean ± SD	119.8 ± 11.5
Diastolic blood pressure (mmHg), mean ± SD	75.9 ± 9.5
Heart rate (BPM), mean ± SD	73.4 ± 12.0
Gender (*n* (%))	
Female	34 (74%)
Male	12 (26%)
Race (*n* (%))	
Black or African American	3 (7%)
White	43 (93%)
Ethnicity (*n* (%))	
Hispanic or Latino	1 (2%)
Not Hispanic or Latino	45 (98%)

**Table 2:** Average daily total intake and macronutrient profile at baseline, week 4, and week 8 (*n*=51, all randomized subjects).

	Energy (kcal)	Protein calories (%)	Carbohydrate calories (%)	Lipid calories (%)	Fiber (g)
	Mean ± SD	Mean ± SD	Mean ± SD	Mean ± SD	Mean ± SD
	Within-group *p* value^a^	Within-group *p* value^a^	Within-group *p* value^a^	Within-group *p* value^a^	Within-group *p* value^a^
Baseline	1,817 ± 423	18.0 ± 4.3	47.0 ± 7.3	34.5 ± 6.8	16.4 ± 5.4
Week 4	1,809 ± 479	17.6 ± 4.0	47.5 ± 7.1	33.8 ± 7.0	16.0 ± 5.8
Week 8	1,780 ± 506	17.6 ± 3.3	47.6 ± 7.1	34.2 ± 5.9	15.5 ± 5.8
Change from baseline to week 4	−7 ± 462	−0.3 ± 4.0	0.6 ± 8.5	−0.7 ± 8.4	−0.4 ± 5.1
	0.849^b^	0.528^b^	0.621^b^	0.542^b^	0.504^b^
Change from baseline to week 8	−51 ± 426	−0.3 ± 4.0	0.7 ± 7.8	−0.4 ± 7.3	−0.9 ± 5.0
	0.325^b^	0.598^b^	0.518^b^	0.755^b^	0.142^b^

*n*: number; SD: standard deviation; kcal: kilocalorie. ^a^Within-group comparisons were made using the paired Student's *t*-test. ^b^Square root transformation required to achieve normality. Probability values ≤0.05 are statistically significant.

**Table 3:** Proportion of Actinobacteria, Bacteroidetes, and Firmicutes in participants supplemented with the anthocyanin-prebiotic blend at baseline (day 0) and end of the study (day 57) (*n*=45).

	Actinobacteria	Bacteroidetes	Firmicutes
	Mean ± SD (*n*)	Mean ± SD (*n*)	Mean ± SD (*n*)
	Median (interquartile range)	Median (interquartile range)	Median (interquartile range)
	Within-group *p* value^a^	Within-group *p* value^a^	Within-group *p* value^a^
Baseline (day 0)	0.085 ± 0.068 (45)	0.138 ± 0.096 (45)	0.749 ± 0.104 (45)
	0.066 (0.095)	0.12 (0.140)	0.765 (0.106)

End of study (day 57)	0.034 ± 0.032 (45)	0.345 ± 0.159 (45)	0.590 ± 0.141 (45)
	0.028 (0.032)	0.341 (0.187)	0.59 (0.195)

Change from baseline to end of the study	−0.052 ± 0.058 (45)	0.208 ± 0.176 (45)	−0.159 ± 0.170 (45)
	−0.038 (0.076)	0.227 (0.195)	−0.18 (0.220)
	<0.001^b^	<0.001^b^	<0.001^b^

*Note*. One participant was unable to provide a fecal sample at the end of the study. *n*: number; SD: standard deviation. ^a^Within-group comparisons were made using the paired Student's *t*-test. ^b^Logarithmic transformation required to achieve normality. Probability values ≤0.05 are statistically significant.

**Table 4:** Bowel habits in participants supplemented with the anthocyanin-prebiotic blend at baseline (day 0) and end of the study (day 57) (*n*=46).

	Bristol Stool Scale (*n*)	Number of bowel movements (*n*/day)	Proportion of bowel movements that were straining to start (%)	Proportion of bowel movements that were straining to stop (%)	Proportion of bowel movements that were incomplete (%)
	Mean ± SD (*n*)	Mean ± SD (*n*)	Mean ± SD (*n*)	Mean ± SD (*n*)	Mean ± SD (*n*)
	Median (interquartile range)	Median (interquartile range)	Median (interquartile range)	Median (interquartile range)	Median (interquartile range)
	Within-group *p* value^a^	Within-group *p* value^a^	Within-group *p* value^a^	Within-group *p* value^a^	Within-group *p* value^a^
Baseline	3.88 ± 0.79 (46)	2.52 ± 1.09 (46)	17.7 ± 22.8 (46)	3.9 ± 10.3 (46)	22.9 ± 27.5 (46)
	3.87 (0.94)	2 (1)	6.7 (25.0)	0 (2.9)	11.4 (34.6)

Change from baseline to week 1	−0.01 ± 0.59 (46)	−0.07 ± 0.88 (46)	1.5 ± 20.7 (46)	−1.1 ± 8.2 (46)	−3.8 ± 23.9 (46)
	0.04 (0.71)	0 (2)	0 (20.4)	0 (0)	0 (19.6)
	0.924	0.709^b^	0.420^b^	0.185^b^	0.191^b^

Change from baseline to week 2	−0.00 ± 0.65 (46)	−0.28 ± 0.89 (46)	−1.1 ± 17.3 (46)	0.9 ± 9.6 (46)	−8.7 ± 22.5 (46)
	−0.03 (0.64)	0 (1)	0 (13.0)	0 (0)	−4 (14.9)
	0.994	0.066^b^	0.083^b^	0.345^b^	<0.001^b^

Change from baseline to week 3	0.10 ± 0.55 (46)	−0.17 ± 0.93 (46)	−2.5 ± 18.0 (46)	3.5 ± 16.1 (46)	−8.7 ± 24.9 (46)
	0.14 (0.63)	0 (1)	0 (16.5)	0 (0)	−3.3 (16.2)
	0.225	0.349^b^	0.089^b^	0.506^b^	<0.001^b^

Change from baseline to week 4	0.11 ± 0.70 (46)	0.04 ± 1.03 (46)	−3.7 ± 19.1 (46)	−1.2 ± 6.3 (46)	−10.9 ± 21.9 (46)
	0.16 (0.82)	0 (2)	−0.8 (10.6)	0 (0)	−2.6 (17.8)
	0.309	0.962^b^	0.004^b^	0.025^b^	<0.001^b^

Change from baseline to week 5	0.11 ± 0.54 (46)	−0.26 ± 0.80 (46)	−5.6 ± 18.0 (46)	0.5 ± 7.5 (46)	−10.7 ± 27.2 (46)
	0.17 (0.64)	0 (1)	−3.9 (13.0)	0 (0)	−4.2 (20.0)
	0.193	0.056^b^	0.002^b^	0.124^b^	<0.001^b^

Change from baseline to week 6	0.17 ± 0.58 (46)	0.02 ± 0.86 (46)	−4.8 ± 14.1 (46)	−1.2 ± 5.8 (46)	−11.6 ± 20.6 (46)
	0.24 (0.65)	0 (0)	−4 (12.8)	0 (0)	−4.5 (21.1)
	0.051	0.891^b^	<0.001^b^	0.167^b^	<0.001^b^

Change from baseline to week 7	0.18 ± 0.53 (46)	−0.15 ± 0.73 (46)	−5.0 ± 16.9 (46)	−2.0 ± 7.4 (46)	−11.6 ± 24.2 (46)
	0.22 (0.67)	0 (1)	0 (14.1)	0 (0)	−6.7 (21.1)
	0.028	0.284^b^	0.002^b^	0.046^b^	<0.001^b^

Change from baseline to week 8	0.18 ± 0.64 (46)	−0.07 ± 0.95 (46)	−5.6 ± 19.1 (46)	−2.2 ± 8.3 (46)	−27 ± 108 (46)
	0.28 (0.81)	0 (1.75)	−1.7 (14.0)	0 (2.9)	−6 (21.7)
	0.063	0.432^b^	<0.001^b^	0.019^b^	<0.001^b^

*n*: number; SD: standard deviation. ^a^Within-group comparisons were made using the paired Student's *t*-test. ^b^Logarithmic transformation required to achieve normality. Probability values ≤0.05 are statistically significant.

**Table 5:** Bloating, abdominal pain, and gas severity in participants supplemented with the anthocyanin-prebiotic blend at baseline (day 0) and end of the study (day 57) (*n*=46).

	Bloating severity (*n*)	Abdominal pain severity (*n*)	Gas severity (*n*)
	Mean ± SD (*n*)	Mean ± SD (*n*)	Mean ± SD (*n*)
	Median (interquartile range)	Median (interquartile range)	Median (interquartile range)
	Within-group *p* value^a^	Within-group *p* value^a^	Within-group *p* value^a^
Baseline (day 0)	2.11 ± 2.07 (46)	1.90 ± 1.83 (46)	2.40 ± 1.96 (46)
	1.93 (2.77)	1.64 (2.74)	2.04 (2.39)

Change from baseline to week 1	−0.10 ± 1.00 (46)	0.09 ± 1.03 (46)	−0.02 ± 1.09 (46)
	−0.14 (1.20)	0 (1.04)	0.07 (0.93)
	0.125^c^	0.914^b^	0.843^b^

Change from baseline to week 2	−0.12 ± 1.15 (46)	0.12 ± 1.15 (46)	−0.02 ± 1.16 (46)
	−0.14 (1.20)	0 (1.16)	−0.11 (1.09)
	0.054^c^	0.972^b^	0.462^b^

Change from baseline to week 3	−0.35 ± 1.21 (46)	−0.18 ± 1.18 (46)	−0.16 ± 1.29 (46)
	−0.24 (0.98)	−0.07 (0.63)	−0.25 (1.21)
	0.005^c^	0.050^b^	0.340^b^

Change from baseline to week 4	−0.61 ± 1.21 (46)	−0.44 ± 1.22 (46)	−0.36 ± 1.11 (46)
	−0.21 (1.21)	−0.14 (1.09)	−0.21 (1.09)
	<0.001^c^	0.006^b^	0.062^b^

Change from baseline to week 5	−0.55 ± 1.34 (46)	−0.35 ± 1.35 (46)	−0.52 ± 1.31 (46)
	−0.14 (1.21)	−0.14 (0.92)	−0.29 (1.32)
	0.001^c^	0.037^b^	0.007^b^

Change from baseline to week 6	−0.59 ± 1.25 (46)	−0.32 ± 1.30 (46)	−0.35 ± 1.38 (46)
	−0.14 (1.05)	−0.11 (0.85)	−0.36 (1.05)
	<0.001^c^	0.031^b^	0.056^b^

Change from baseline to week 7	−0.52 ± 1.24 (46)	−0.33 ± 1.16 (46)	−0.34 ± 1.22 (46)
	−0.14 (1.11)	−0.04 (0.64)	−0.25 (0.71)
	0.002^c^	0.014^b^	0.043^b^

Change from baseline to week 8	−0.42 ± 1.01 (46)	−0.28 ± 0.98 (46)	−0.26 ± 1.07 (46)
	−0.11 (1.18)	0 (0.70)	−0.21 (0.84)
	<0.001^c^	0.014^b^	0.035^b^

*n*: number; SD: standard deviation. ^a^Within-group comparisons were made using the paired Student's *t*-test. ^b^Within-group comparisons were made using the signed-rank test. ^c^Square root transformation required to achieve normality. Probability values ≤0.05 are statistically significant.

## Data Availability

The microbiome data are available from the corresponding author upon request.

## Abbreviations

ALT: Alanine aminotransferase
AST: Aspartate aminotransferase
BSS: Bristol Stool Scale
CBC: Complete blood count
DP: Degrees of polymerization
FOSs: Fructooligosaccharides
GGT: Gamma-glutamyl transferase
scFOSs: Short-chain fructooligosaccharides
HbA1c: Hemoglobin A1c.

## Supplementary Materials

Supplementary Materials The study data file in supplementary materials contains complete subject's demographics, vital signs, product compliance, labs, bowel diary records, abdominal discomfort questionnaire, 3-day food record, and all adverse events.
