# Supplementary material for: Efficacy of an Anthocyanin and Prebiotic Blend on Intestinal Environment in Obese Male and Female Subjects
Source: J Nutr Metab. 2018 Sep 13;2018:7497260. doi: 10.1155/2018/7497260 (PMC6158948; doi:10.1155/2018/7497260)
Supplement: Supplementary Materials — The study data file in supplementary materials contains complete subject's demographics, vital signs, product compliance, labs, bowel diary records, abdominal discomfort questionnaire, 3-day food record, and all adverse events. [file 7497260.f1.zip › Supplemental Document 1. Bowel movement diary.docx]

| Date: | | | | | | | |
| --- | --- | --- | --- | --- | --- | --- | --- |
| How many glasses of water did you consume today?  ____________ glasses  *One glass of water equals approximately 8oz (250 ml)* | | | | Comments/Therapies used and reason for use: | | | |
| **For each bowel movement you experienced today record the time for question 1 and circle Yes, No (Y/N) or N/A for questions 2-7.**  **For question 5 please refer to the Bristol Stool Chart and record the type that best describes the bowel movement.**  **If you do not have any bowel movements, circle 0 and complete question 2, 4, 7, and 8.** | | | | | | | |
| **Bowel Movement Number**  *If required, additional movements and details can be added to the back of this questionnaire* | | | | | | | |
|  | 0 | 1 | 2 | | 3 | 4 | 5 |
| 1. Time of bowel movement (hh:mm) | N/A | ___:___ | ___:___ | | ___:___ | ___:___ | ___:___ |
| 1. Straining to start defecation? | Y / N  or  N/A | Y / N | Y / N | | Y / N | Y / N | Y / N |
| 1. Straining to stop defecation? | N/A | Y / N | Y / N | | Y / N | Y / N | Y / N |
| 1. Feeling of incomplete defecation? | Y / N  Or  N/A | Y / N | Y / N | | Y / N | Y / N | Y / N |
| 1. Bristol stool form type (1-7) | N/A |  |  | |  |  |  |
| 1. Describe the stool color. | N/A |  |  | |  |  |  |
| 1. Were laxatives, enemas, or suppositories used in the 24 hours prior to the bowel movement? | Y / N | Y / N | Y / N | | Y / N | Y / N | Y / N |
| 1. For Females: Do you feel that your bowel movements today were affected by your menstrual cycle? Y / N | | | | | | | |
