# Supplementary material for: Efficacy of an Anthocyanin and Prebiotic Blend on Intestinal Environment in Obese Male and Female Subjects
Source: J Nutr Metab. 2018 Sep 13;2018:7497260. doi: 10.1155/2018/7497260 (PMC6158948; doi:10.1155/2018/7497260)
Supplement: Supplementary Materials — The study data file in supplementary materials contains complete subject's demographics, vital signs, product compliance, labs, bowel diary records, abdominal discomfort questionnaire, 3-day food record, and all adverse events. [file 7497260.f1.zip › Supplemental Document 2. Abdominal discomfort questionnaire.docx]

Please mark a cross (X) on the scales below to accurately indicate the severity of the symptoms you have experienced today.

1. Please indicate the level of **abdominal discomfort** you experienced today:

None O-----O-----O-----O-----O-----O-----O-----O-----O-----O-----O Worst

0 1 2 3 4 5 6 7 8 9 10

1. Do you feel your **abdominal discomfort** is related to food you consumed? YES NO
2. Please indicate the level of **abdominal bloating** you experienced today:

None O-----O-----O-----O-----O-----O-----O-----O-----O-----O-----O Worst

0 1 2 3 4 5 6 7 8 9 10

1. Do you feel your **abdominal bloating** is related to food you consumed? YES NO
2. Please indicate the level of **gas (flatulence)** you experienced today:

None O-----O-----O-----O-----O-----O-----O-----O-----O-----O-----O Worst

0 1 2 3 4 5 6 7 8 9 10

1. Do you feel your **gas (flatulence)** is related to food you consumed? YES NO

FOR FEMALES ONLY:

1. Do you feel your **abdominal discomfort** is related to your menstrual cycle? YES NO
2. Do you feel your **abdominal bloating** is related to your menstrual cycle? YES NO
3. Do you feel your **gas (flatulence)** is related to your menstrual cycle? YES NO
